# Submucosal tunnel endoscopic resection for removal of deep-seated rectal gastrointestinal stromal tumor in the muscularis propria

**DOI:** 10.1055/a-2466-9742

**Published:** 2024-12-04

**Authors:** Shaotong Wang, Gangping Li, Jun Song

**Affiliations:** 1 36630 Gastroenterology, Huazhong University of Science and Technology Tongji Medical College Union Hospital, Wuhan, China

Treatment of rectal gastrointestinal stromal tumors (GISTs) is complex, and surgical resection is recommended regardless of tumor size 1 . No definitive method has yet been established.

A 41-year-old woman underwent a colonoscopy at our hospital during a routine health screening, revealing a hemispherical protrusion approximately 1.2 cm in diameter located just above the dentate line of the distal rectum ( Fig. 1 ). Endoscopic ultrasound showed the lesion measuring approximately 12.3 mm by 9.4 mm, and originating from the muscularis propria ( Fig. 2 ). The patient underwent submucosal tunnel endoscopic resection (STER) under propofol anesthesia ( Video 1 ). Initially, submucosal injection of methylene blue was performed for lifting, and a golden knife (Nanwei Medical Technology, Nanjing, China) was employed to incise the external mucosa and establish the tunnel entry ( Fig. 3 ). Since the tumor was not detected in the superficial layer of the muscularis propria, the layer was incised to expose the tumor. The deep white tumor in the muscularis propria was completely resected using the golden knife ( Fig. 4 ). The procedure was completed within 45 minutes. Pathological results indicated a rectal GIST, with a risk of recurrence classified as intermediate. Immunohistochemistry showed: CD117 (+), CD34 (+), DOG-1 (+), SMA (–), desmin (–), S100 (–), SOX10 (–), ALK (–), SDHB (+), Ki67 (Li: 5%) ( Fig. 5 ). The patient experienced no postoperative complications, and adjuvant therapy with imatinib will be considered based on the results of genetic testing (KIT and FDGRRA genes).

**Fig. 1:**
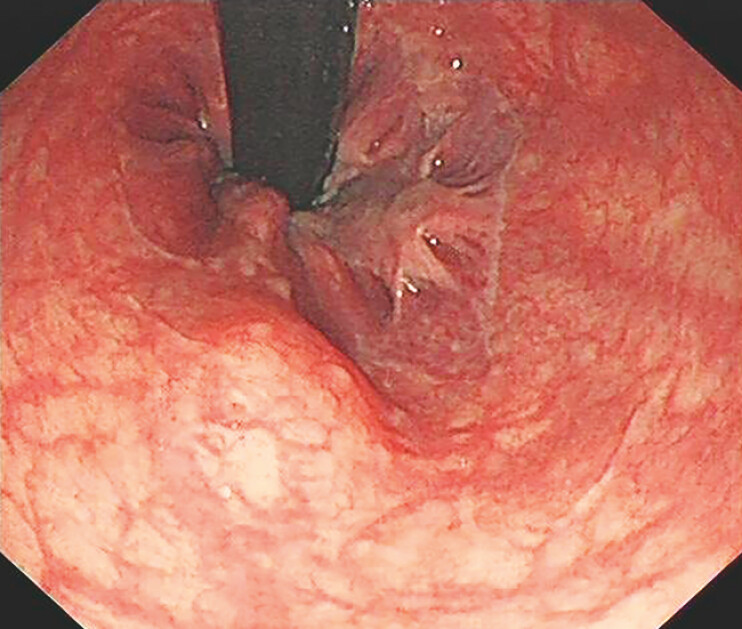
Routine screening colonoscopy in a 41-year-old woman revealed a submucosal protrusion approximately 1.2 cm in diameter located just above the dentate line.

**Fig. 2:**
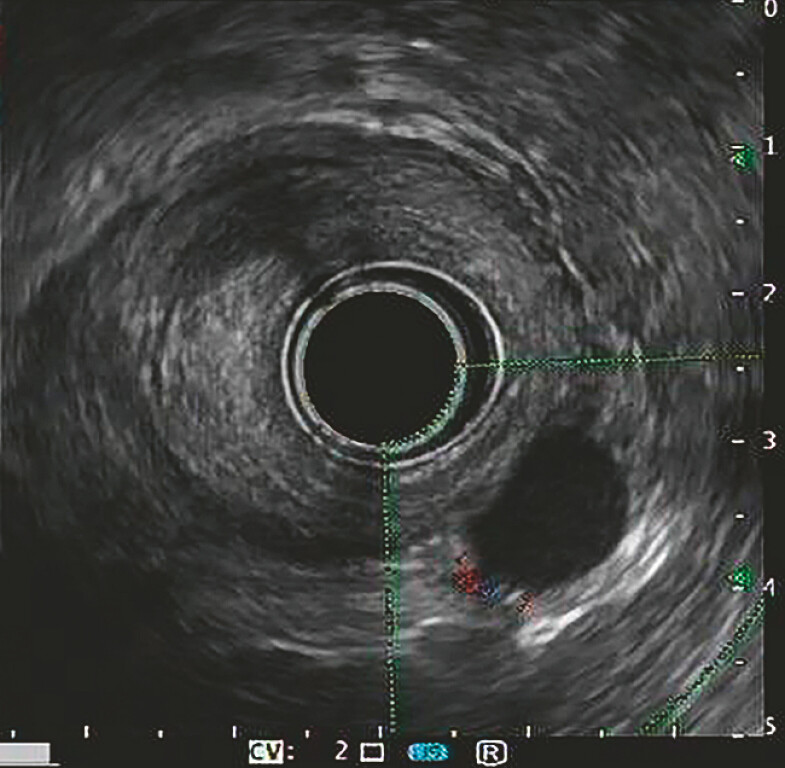
Endoscopic ultrasound imaging showed that the lesion originated from the muscularis propria and was located deep within it.

**Fig. 3:**
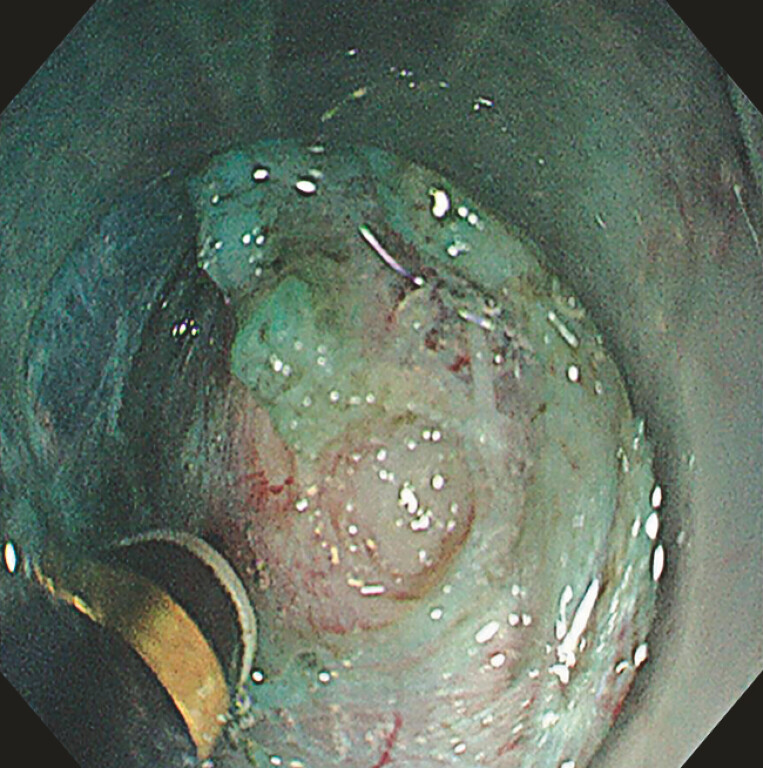
The submucosal tunnel provided good exposure of the tumor.

**Fig. 4:**
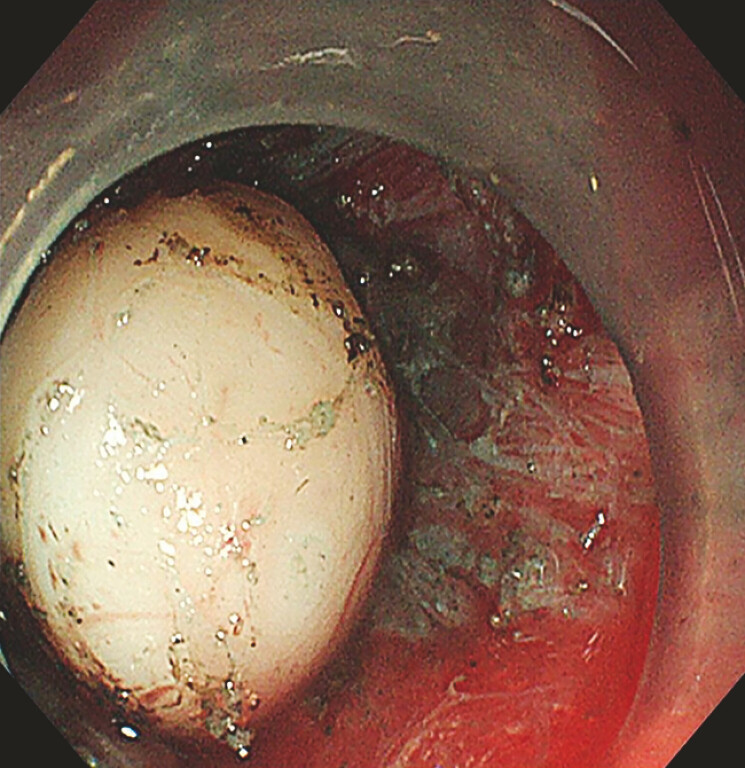
Complete resection of the tumor.

**Fig. 5:**
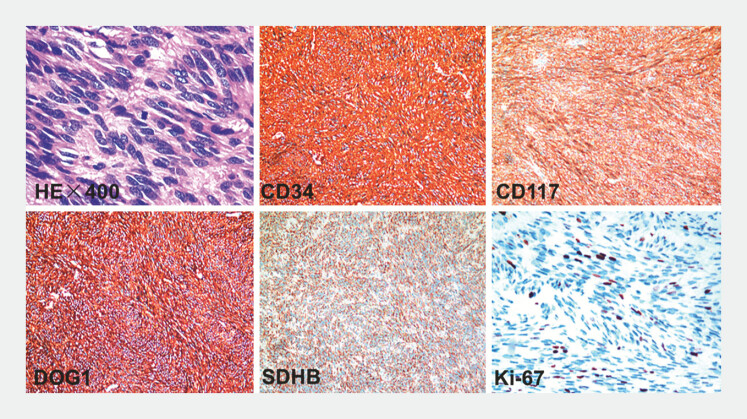
Pathological findings confirmed a rectal gastrointestinal stromal tumor (GIST).

Submucosal tunnel endoscopic resection (STER) of a rectal gastrointestinal stromal tumor (GIST).Video 1

Endoscopy_UCTN_Code_TTT_1AQ_2AD_3AZ

## Notes

Endoscopy E-Videos https://eref.thieme.de/e-videos
*E-Videos* is an open access online section of the journal *Endoscopy* , reporting on interesting cases and new techniques in gastroenterological endoscopy. All papers include a high-quality video and are published with a Creative Commons CC-BY license. Endoscopy E-Videos qualify for HINARI discounts and waivers and eligibility is automatically checked during the submission process. We grant 100% waivers to articles whose corresponding authors are based in Group A countries and 50% waivers to those who are based in Group B countries as classified by Research4Life (see: https://www.research4life.org/access/eligibility/ ). This section has its own submission website at https://mc.manuscriptcentral.com/e-videos .
